# CD19 + CD21^lo/neg^ cells are increased in systemic sclerosis-associated interstitial lung disease

**DOI:** 10.1007/s10238-021-00745-5

**Published:** 2021-08-10

**Authors:** Erin M. Wilfong, Katherine N. Vowell, Kaitlyn E. Bunn, Elise Rizzi, Narender Annapureddy, Rosemarie B. Dudenhofer, April Barnado, Rachel H. Bonami, Joyce E. Johnson, Leslie J. Crofford, Peggy L. Kendall

**Affiliations:** 1 Division of Allergy, Pulmonary, and Critical Care Medicine, Vanderbilt University Medical Center, Nashville, TN 37232, USA; 2 Division of Rheumatology and Immunology, Vanderbilt University Medical Center, Nashville, TN, USA; 3 Deparment of Pathology, Microbiology, and Immunology, Vanderbilt University Medical Center, Nashville, TN, USA; 4 Vanderbilt Institute for Infection, Immunology, and Inflammation, Nashville, TN, USA; 5 Division of Allergy and Immunology, Department of Medicine, Washington University School of Medicine, 660 S. Euclid Ave, Campus Box 8122, St. Louis, MO 63110, USA

**Keywords:** Systemic sclerosis, Interstitial lung disease, CD21lo B cells, Mass cytometry

## Abstract

Interstitial lung disease (ILD) represents a significant cause of morbidity and mortality in systemic sclerosis (SSc). The purpose of this study was to examine recirculating lymphocytes from SSc patients for potential biomarkers of interstitial lung disease (ILD). Peripheral blood mononuclear cells (PBMCs) were isolated from patients with SSc and healthy controls enrolled in the Vanderbilt University Myositis and Scleroderma Treatment Initiative Center cohort between 9/2017–6/2019. Clinical phenotyping was performed by chart abstraction. Immunophenotyping was performed using both mass cytometry and fluorescence cytometry combined with t-distributed stochastic neighbor embedding analysis and traditional biaxial gating. This study included 34 patients with SSc-ILD, 14 patients without SSc-ILD, and 25 healthy controls. CD21^lo/neg^ cells are significantly increased in SSc-ILD but not in SSc without ILD (15.4 ± 13.3% vs. 5.8 ± 0.9%, *p* = 0.002) or healthy controls (5.0 ± 0.5%, *p* < 0.0001). While CD21^lo/neg^ B cells can be identified from a single biaxial gate, tSNE analysis reveals that the biaxial gate is comprised of multiple distinct subsets, all of which are increased in SSc-ILD. CD21^lo/neg^ cells in both healthy controls and SSc-ILD are predominantly tBET positive and do not have intracellular CD21. Immunohistochemistry staining demonstrated that CD21^lo/neg^ B cells diffusely infiltrate the lung parenchyma of an SSc-ILD patient. Additional work is needed to validate this biomarker in larger cohorts and longitudinal studies and to understand the role of these cells in SSc-ILD.

## Introduction

Systemic sclerosis (SSc) is a rare autoimmune condition afflicting between 7 and 489 per million people worldwide [1]. Interstitial lung disease (ILD), a major cause of morbidity and mortality, is seen in 15–38% of SSc patients [2–5] with a higher burden of lung involvement among African Americans patients [6]. Since the introduction of captopril for the management of SSc renal crisis, pulmonary fibrosis has become the leading cause of SSc-related mortality [7, 8]. ILD is also a significant cause of morbidity, and dyspnea significantly affects health-related quality of life measures independent of overall disease severity [9, 10].

Ideally, a biomarker would be available for SSc-ILD that would correlate with the development, presence, and severity of SSc-ILD while also giving insights into the underlying disease pathophysiology. To date, such a biomarker has been elusive. Protein biomarkers surfactant protein D and Krebs von den Lungen-6 are markers of lung inflammation and injury not only in SSc-ILD [11] but also in acute respiratory distress syndrome [12, 13], chronic obstructive pulmonary disease [14], and acute exacerbation of idiopathic pulmonary fibrosis [15]. Cytokines and chemokines have also been investigated. While interleukin-6 (IL-6) predicts early functional decline and mortality in patients with mild ILD, it does not have a role in more severe ILD [16]. CXCL4 is associated with ILD and a clinically significant decline in % predicted forced vital capacity (FVC), but this biomarker is not specific for ILD [17].

A limitation of protein-based biomarkers is that they do not give insight into cellular aberrancies driving the underlying disease process. To that end, researchers have also investigated cell subsets that may correlate with the presence of ILD. A collagen producing CD14 + monocyte is increased in SSc-ILD compared to healthy controls [18], but an SSc without ILD comparator group was not included. IL-22 producing CD4 + T cells are increased in SSc-ILD compared to SSc without ILD, but significant overlap remained between the groups [19]. Increased frequency of *T*_reg_ cells correlates with reduced diffusing capacity of the lungs for carbon monoxide (DLCO), but DLCO is affected both in pulmonary arterial hypertension (PAH) and ILD. This work did not investigate the association with FVC, which would be more specific for ILD [20]. While no correlation between B cell subsets and SSc-ILD has been reported to date, serumfree light chains were increased in one SSc-ILD cohort [21]. Thus, it is plausible that a B cell subset might drive SSc-ILD given the known infiltration of B cells in SSc-ILD [22] and open-label trials of rituximab in SSc-ILD [23, 24].

The purpose of this study was to investigate if a B cell population might serve as a cellular biomarker for SSc-ILD using a combination of targeted gating of known autoimmune-prone subsets [25–28] and minimally supervised analyses using mass cytometry by time-of-flight (CyTOF).

## Patients and methods

### Patient enrollment and clinical phenotyping

Institutional Review Board approval was obtained. Patients with suspected SSc, idiopathic inflammatory myopathies, mixed connective tissue disease, or interstitial pneumonia with autoimmune features were eligible for referral to the MYSTIC cohort by their treating provider in the outpatient pulmonary, thoracic surgery, or rheumatology clinics, the inpatient rheumatology or pulmonary consulting services, or by their critical care provider in the intensive care unit (VUMC IRB 141415). Patients included in these investigations were enrolled between 9/17/2017 and 6/1/2019 and met the 2013 ACR/EULAR criteria for systemic sclerosis [29]. Individuals enrolling as healthy controls completed a health questionnaire to verify a negative review of systems and no known personal or family history of autoimmunity.

Clinical phenotyping was performed by clinical chart abstraction to estimate the date of first non-Raynaud’s symptom onset, degree of skin thickening, and active medications. Disease modifying anti-rheumatic drugs were defined as azathioprine, mycophenolate mofetil, tocilizumab, methotrexate, leflunomide, and tofacitinib. Serologic data collected included ANA titer and pattern, rheumatoid factor (RF), anti-cyclic citrullinated peptide, anti-Scl70, anti-RNA polymerase III, and anti-Pm/Scl. Pulmonary phenotyping was performed using chart abstraction of computerized tomography (CT) scan, PFTs, echocardiogram, and right heart catheterization (RHC) reports. Patients were classified as having interstitial lung disease if the radiologist determined that fibrosis or interstitial lung disease was present on CT scan. Radiographic features of ILD including ground glass opacities, traction bronchiectasis, increased reticulations, and honeycombing were abstracted from the reading radiologist’s report. Patients were classified as having PAH if mean pulmonary artery pressure on RHC > 20 mmHg with normal pulmonary capillary wedge pressure or an echocardiogram demonstrated right ventricular systolic pressure > 60 mmHg in the absence of RHC. Clinical phenotyping data are reported as mean ± standard deviation unless otherwise indicated.

### Peripheral blood mononuclear cell (PBMC) isolation

SSc patients (*n* = 48) and healthy controls (*n* = 25) underwent venipuncture during which 32 mL of blood was collected in sodium heparin CPT tubes (BD Biosciences, San Jose CA), and PBMCs were isolated according to the manufacturer’s directions. Red blood cell lysis was performed using Gibco ACK Buffer (ThermoFisher). Purified PBMCs were counted using Ac-T Diff Hematology Analyzer (Beckman Coulter, Indianapolis, IN) and cryopreserved at 4–5 × 10^6^ PBMCs/mL in GemCell heat inactivated fetal bovine serum (Gemini Bioproducts) using controlled rate cell freezing containers.

### Cytometry by time of flight (CyTOF) data acquisition

Individual PBMC cryotubes were thawed in 10 mL warm PBS w/o calcium or magnesium (Gibco, Life Technologies, Grand Island, NY), pelleted by centrifugation and washed twice with 10 mL PBS. Cells were transferred to a 96-well plate for staining. Cells were incubated first with a viability reagent (200 nM cisplatin-198, Fluidigm) in phosphate-buffered saline (PBS) for 5 min; the reaction was quenched using 125 μL PBS/1% bovine serum albumin (BSA). Clones used for staining are shown in table S1. Cells were washed twice with PBS/1% BSA. Cells were resuspended in 100 μL surface stain master mix and incubated for 30 min at room temperature (RT). 50 μL of PBS/1%BSA was added to each well, and the cells were pelleted by centrifugation and washed once with PBS/1% BSA. The cells were resuspended in 100 μL secondary surface master mix for 30 min. Cells were pelleted by centrifugation and washed once with PBS without BSA. Cells were then fixed with 100 μL 1.6% paraformaldehyde, incubated for 20 min and washed with PBS. Cells were permeabilized using Ebioscience FoxP3 fix/perm buffer (Thermofisher) for 45 min at RT. Cells were pelleted by centrifugation and washed in fix/perm buffer prior to resuspension in 100 μL primary intracellular master mix for 30 min at RT. Cells were pelleted by centrifugation and washed with fix/perm buffer before resuspending in secondary intracellular master mix for 30 min at RT. Cells were pelleted by centrifugation, washed with fix/perm buffer and resuspended in 1.6% paraformaldehyde with 6.25 nM intercalator overnight at 4 degrees. Data were collected within 72 h of staining.

On the day of data acquisition, cells were washed twice with PBS without calcium or magnesium and once with 2 mL milliQ water. Cell concentration was adjusted to ~ 500,000 cells/mL with milliQ water, and 10% volume of equilibration beads (Fluidigm Sciences, Sunnyvale, CA) was added to the cell suspension. Cells were filtered immediately before injection into the mass cytometer using a 35 μm nylon mesh cell-strainer cap (BD Biosciences). Data were acquired using a CyTOF Helios (Fluidigm Sciences, Sunnyvale, CA) and CyTOF software (version 6.7.1014) at the Vanderbilt University Medical Center Mass Cytometry Center of Excellence. Dual count calibration and noise reduction were applied during the acquisition; 100,000–400,000 events were collected per sample.

### Flow cytometry

Six representative SSc-ILD were selected based on having the most available biospecimen in the absence of disease modifying anti-rheumatic drugs (DMARDs). Samples were age/sex matched 1:1 with healthy controls. Cryopreserved PBMCs were thawed in 10 mL of PBS w/o calcium or magnesium, pelleted by centrifugation, resuspended in fluorescence-activated cell sorting (FACS) buffer containing fetal bovine serum and sodium azide and washed once more with FACS buffer. Cells were transferred to a 96-well plate for staining. After incubation with 5 μL Fc Block (BD Biosciences, San Jose, CA) in 45μL FACS buffer for 10 min on ice, surface master mix (Table S2) was added for 30 min on ice without centrifugation prior to adding Live/Dead 700 for an additional 5 min. Cells were pelleted by centrifugation, washed twice with FACS buffer, and resuspended in eBioscience FoxP3 Fix/Perm solution (ThermoFisher Scientific, USA) for 30 min, pelleted by centrifugation, and washed with FoxP3 Fix/Perm buffer. After pelleting by centrifugation, cells were resuspended in intracellular master mix in FoxP3 Fix/Perm buffer for 30 min at RT, pelleted by centrifugation and washed twice with FoxP3 Fix/Perm buffer prior to transferring to FACS tubes for data acquisition. All data were acquired on a BD LSRII Fortessa instrument.

### Data analysis

Mass cytometry FCS files underwent Fluidigm bead normalization and were analyzed using Cytobank software per established methods [30]. Traditional biaxial gating identified live B cells (CD19^+^CD3^−^) and previously described biaxial subsets. tSNE plots were generated using equal B cell sampling, 7500 iterations, perplexity of 75, theta of 0.3, and 32 parameters (Table S3). Figure S1 shows the resultant tSNE heatmap for all parameters. Fluorescence cytometry data were analyzed using FlowJo version 9.9.6. Population statistics were exported from CytoBank or FlowJo and analyzed with GraphPad Prism software (GraphPad, La Jolla, CA, USA) to determine mean, standard error of the mean, Kruskal–Wallis p value, Mann–Whitney U tests, and Spearman’s r correlation as indicated.

### Immunohistochemical (IHC) staining

IHC was performed on the one patient for whom lung tissue was available following a lung transplant. A control specimen from surgically resected post-obstructive pneumonia was used as a comparator. Slides of 10% neutral-buffered formalin fixed paraffin embedded tissue previously obtained for clinical purposes were utilized for these investigations. IHC staining for CD21 was performed through the VUMC clinical diagnostic histology core. IHC staining for CD19 was done at Phenopath (Seattle, WA, USA).

## Results

### Patient characteristics

Forty-eight SSc patients meeting 2013 ACR/EULAR criteria for systemic sclerosis and 25 healthy controls were included in this study. Basic demographic and clinical information for all SSc patients compared to healthy controls are shown in Table 1. Basic demographic in clinical information for SSc-ILD and SSc patients without ILD is shown in Table S4. Detailed clinical and pulmonary phenotyping is shown in Tables S5 and S6, respectively. Patients were older than healthy controls (59.4 ± 15.4 vs. 49.4 ± 11.9, *p* < 0.0001). There was no difference in the sex or race/ethnicity of healthy controls compared with SSc patients. Thirty percent of patients took a disease modifying anti-rheumatic drug at enrollment, while 37.5% of patients had not taken any SSc medications in the 6 months prior to enrollment. No patients were treated with anti-fibrotic therapy.

### SSc patients have a decreased frequency of non‑class‑switched memory B cells compared to healthy controls

**Fig. 1:**
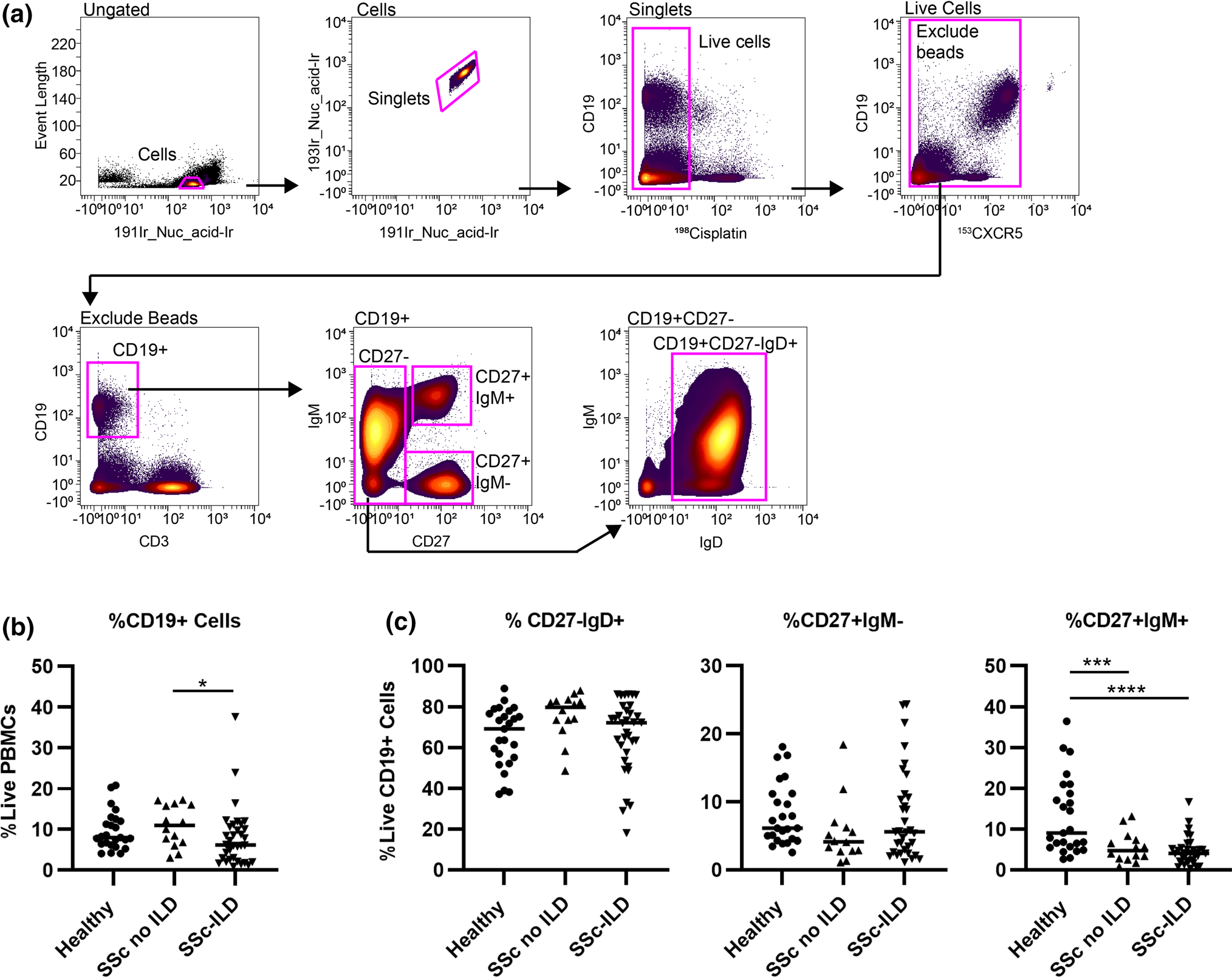
Frequency of CD19^+^ populations in SSc patients with ILD (*n* = 34), SSc patients without ILD (*n* = 14), and healthy controls (*n* = 25). **a** Biaxial gating scheme of CyTOF data for a representative healthy control to identify major subsets of circulating peripheral CD19 + B cells: naïve (CD27^−^/IgM^+^/IgD^+^), class-switched memory (CD27^+^/IgM^−^/IgD^−^), and non-class-switched memory (CD27^+^/IgM^+^). **b** Quantification of total circulating CD19^+^ frequency. **c** Quantification of naïve, class switched, and non-class-switched B cell frequencies. Statistical significance was determined initially with a Kruskal–Wallis across all subgroups followed by Mann–Whitney *U* tests for comparisons between groups if Kruskal–Wallis *p* < 0.05. *p < 0.05, **p < 0.01, ***p < 0.001, ****p < 0.0001

Biaxial gating was utilized to identify all major classes of circulating B cells (Fig. 1). Frequencies of major circulating subsets of B cells are shown in Table 2. While SSc-ILD patients had a slightly lower frequency of CD19^+^ cells compared to SSc patients without ILD, there was no difference between SSc-ILD patients and healthy controls. Non-class-switched memory B cells were decreased in SSc patients with or without ILD patients compared to healthy controls. There was no difference in the frequency of naïve or class-switched memory between subgroups. There was no difference in the frequency of CD19^+^ cells in SSc-ILD patients who were being treated versus those not on therapy at the time of enrollment (7.9 ± 1.8% vs. 7.8 ± 1.8%, *p* = 0.33).

### CD21^lo/neg^ B cells are increased in SSc patients with ILD

**Fig. 2:**
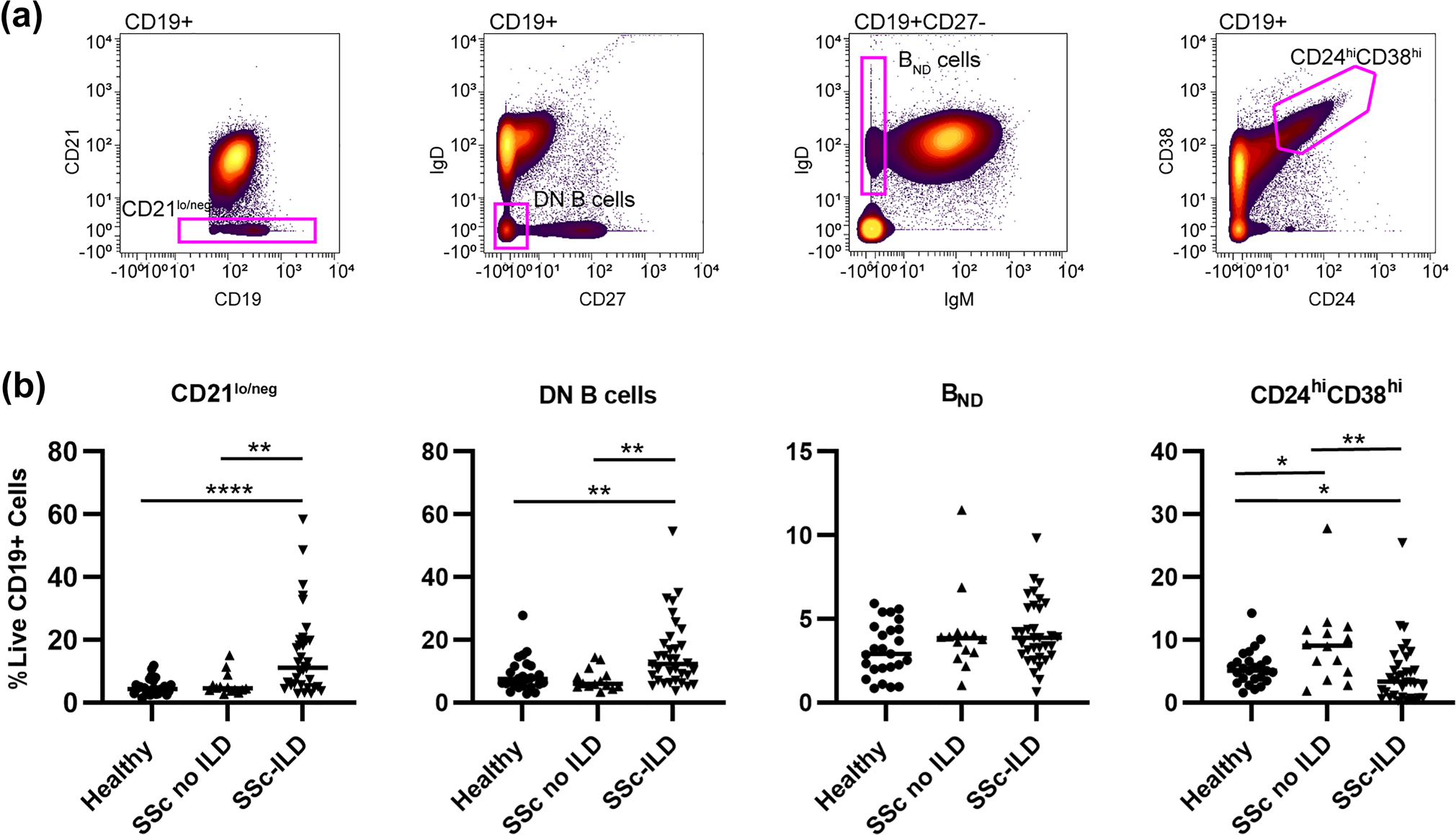
Biaxial gating of previously described autoimmune-prone subsets applied to systemic sclerosis. **a** Biaxial gating schemes for CyTOF data to identify previously described autoimmune-prone B cell subsets are shown for a representative patient. **b** Quantification of autoimmune-prone subsets in SSc-ILD patients (*n* = 34), SSc patients without ILD (*n* = 14) and healthy controls (*n* = 25). Statistical significance was determined initially with a Kruskal–Wallis across all subgroups followed by Mann–Whitney *U* tests for comparisons between groups if Kruskal–Wallis *p* < 0.05. *p < 0.05, **p < 0.01, ***p < 0.001, ****p < 0.0001

**Fig. 3:**
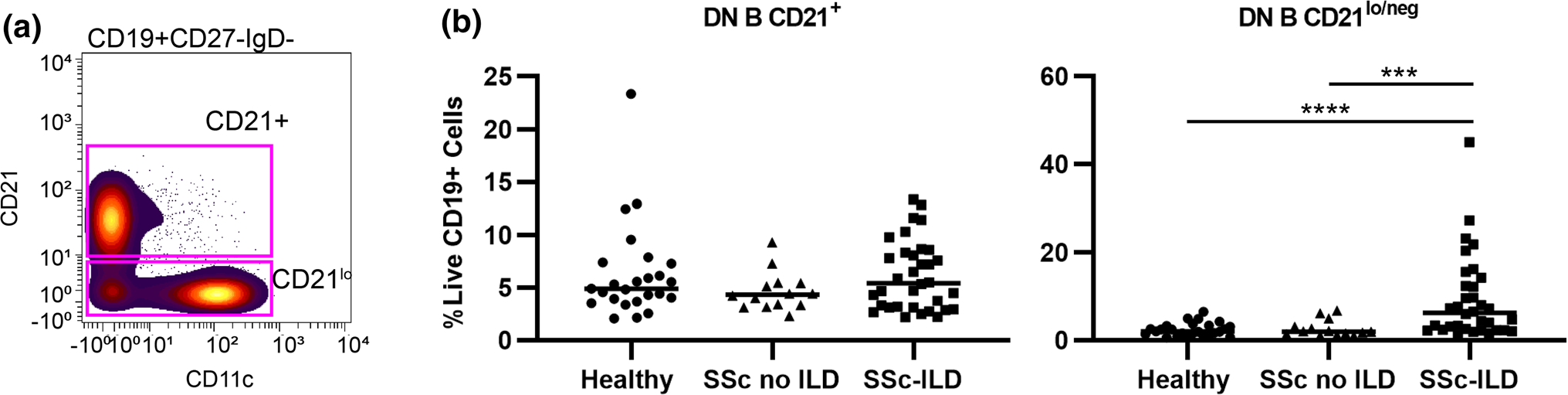
CD21^lo/neg^ subset of DN B cells drives the increased frequency in SSc-ILD. **a** Biaxial gating schemes of CD21^+^ and CD21^lo/neg^ DN B cells shown for a representative SSc-ILD patient. **b** Quantification of CD21^+^ DN B and CD21^lo/neg^ DN B cells for SSc-ILD patients (*n* = 34), SSc patients without ILD (*n* = 14) and healthy controls (*n* = 25). Statistical significance was determined initially with a Kruskal–Wallis across all subgroups followed by Mann–Whitney U tests for comparisons between groups if Kruskal–Wallis *p* < 0.05. *p < 0.05, **p < 0.01, ***p < 0.001, ****p < 0.0001

Next, we investigated if previously published autoimmune-prone B cell subsets, such as CD21^lo/neg^ cells [25], DN B cells (CD27IgD^−^) [31], B_ND_ cells (CD27^−^IgM^−^IgD^+^) [28], or CD24^hi^CD38^hi^ transitional cells [27], were increased in SSc-ILD (Fig. 2 and Table 2). Comparisons between all SSc patients and healthy controls are shown in Supplemental Figure S2. SSc-ILD patients had an increased frequency of CD21^lo/neg^ B cells compared to both healthy controls and SSc patients without ILD. There was no difference between SSc patients without ILD and healthy controls. There was no difference in the frequency of CD21^lo/neg^ cells in diffuse versus limited cutaneous SSc (Supplemental Figure S3a). Examining the relationship between CD21^lo/neg^ cells and PAH is confounded as ILD is an independent risk factor for the development of PAH [32]. While SSc-PAH patients had an increased frequency of CD21^lo/neg^ cells compared to SSc patients without PAH (18.4 ± 3.6% vs. 9.5 ± 1.7%, *p* = 0.0002), there is no difference between SSc-PAH and SSc patients with ILD but not PAH (18.4 ± 3.6% vs. 12.5 ± 2.6%, *p* = 0.23) (Supplemental Figure S3b–c). Additionally, 15/17 SSc-PAH patients in this cohort also had concomitant ILD. While DN B cells were also found to be increased in SSc-ILD compared to SSc-without ILD and healthy controls (Fig. 2b), the DN B population is comprised of both CD21 and CD21^lo/neg^ cells. Only CD21^lo/neg^ DN B cells were increased in SSc-ILD; there was no difference in CD21^+^ DN B cells (Fig. 3). CD24^hi^CD38^hi^ transitional cells were decreased in SSc-ILD compared to healthy controls and SSc patients without ILD. No differences were observed in B_ND_ cell frequency.

**Fig. 4:**
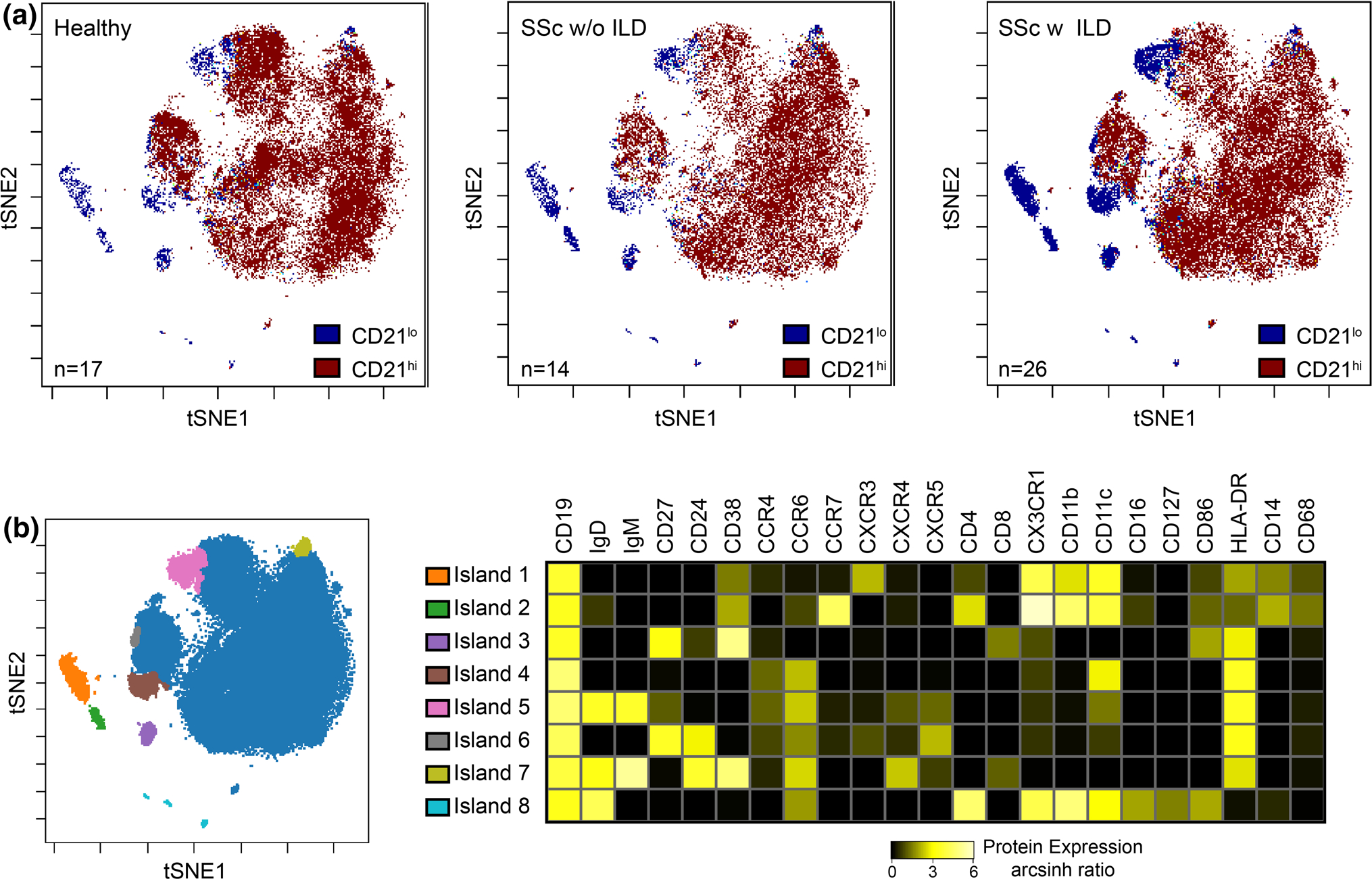
Significant heterogeneity exists within the CD21^lo/neg^ population, and there is no single driving population. **a** tSNE dot plots were generated using an equal number of CD19^+^ B cells for 26 SSc patients with ILD, 14 SSc patients without ILD, and 17 healthy control analyzed with the same antibody lot. The dot density is increased in all CD21^lo^ islands in SSc-ILD compared with SSc patients without ILD and healthy controls. **b** Overlaid figure dimension showing 8 distinct islands of CD21^lo/neg^ B cells accompanied by a heat map of transformed ratio of median expression by table minimum

To further probe the heterogeneity of the CD21^lo/neg^ subset, 40 SSc patients and 17 healthy controls analyzed using the same antibody lot were subjected to tSNE analysis (Fig. 4). Batch effects precluded the inclusion of the remaining samples. tSNE analysis revealed that CD21^lo/neg^ B cells are highly heterogeneous and comprise multiple distinct subsets. The increased frequency of CD21^lo/neg^ B cells arises from an increase in each of these distinct populations; there is not one subgroup of CD21^lo/neg^ cells that drives global increase in CD21^lo/neg^ cells.

Spearman correlations were performed to ascertain if the frequency of circulating CD21^lo/neg^ cells correlated with ILD severity or age. There was no correlation with the circulating number of CD21^lo/neg^ cells with FVC (Spearman *r* = 0.044, *p* = 0.86). There was a low to moderate correlation between age and the frequency of CD21^lo/neg^ cells for all SSc patients (Spearman 0.36, *p* = 0.01). There was also no difference in the frequency of CD21^lo/neg^ cells in SSc-ILD patients on DMARD therapy compared to patients taking no medications (15.1 ± 12.9% vs. 15.7% ± 14.4%, *p* = 0.99).

### CD21^lo/neg^ B cells are predominantly tBET positive but do not have intracellular CD21 expression

**Fig. 5:**
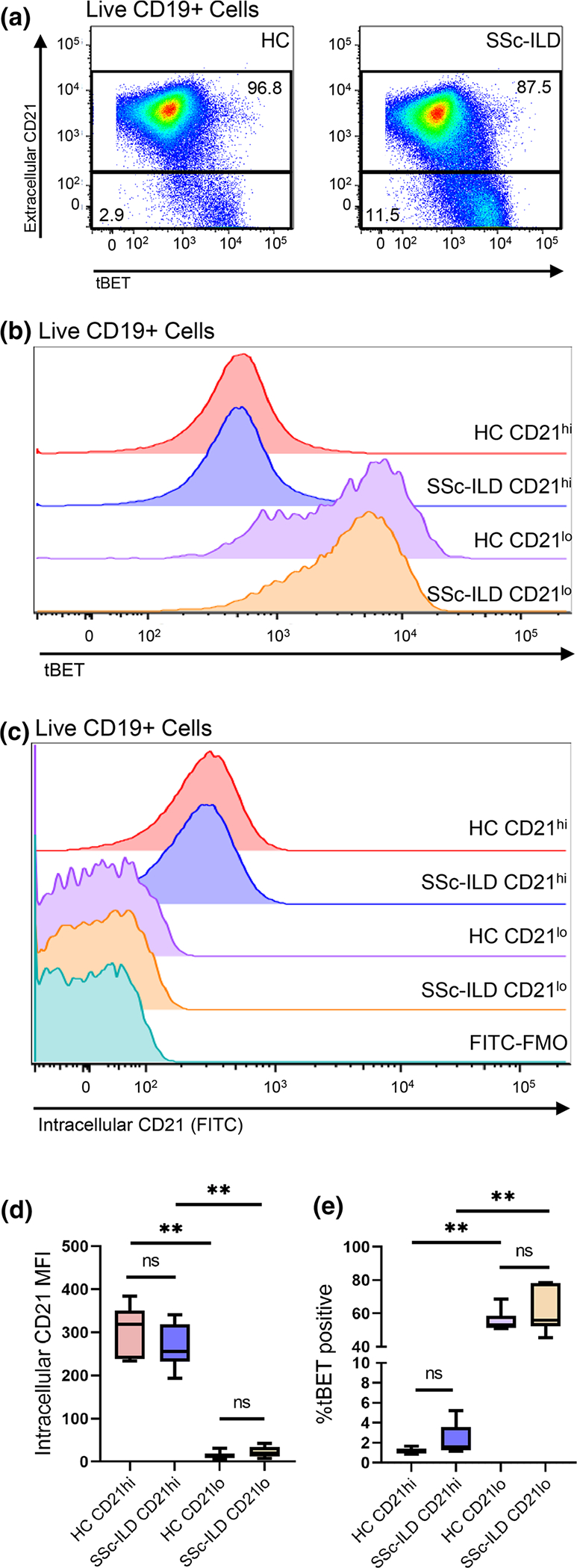
CD21^lo/neg^ B cells are predominantly tBET positive but do not have intracellular CD21 expression. **a** CD21^lo/neg^ cells have increased tBET expression compared to CD21^+^ cells in both SSc-ILD (*n* = 6) and healthy controls (*n* = 6). **b** Concatenated histogram showing tBET expression in both CD21^+^ and CD21^lo/neg^ B cells for both healthy controls and SSc-ILD patients. **c** Concatenated histogram showing that while some CD21 is detected intracellularly in CD21^+^ cells, CD21^lo/neg^ B cells do not have detectable intracellular CD21. **d** The intracellular CD21 mean fluorescence intensity (MFI) is increased in CD21^+^ versus CD21^lo/neg^ cells in both SSc-ILD (266.8 ± 21.3 vs. 22.0 ± 12.8, *p* = 0.002) and healthy controls (306.0 ± 24.2 vs. 14.14 ± 3.6, *p* = 0.002), but there was no difference in intracellular CD21 MFI in SSc-ILD compared to healthy CD21^lo/neg^ cells (22.4 ± 5.2 vs. 14.1 ± 3.6, *p* = 0.26). **e** The percent of tBET positive cells is increased in CD21^lo/neg^ cells compared to CD21^+^ cells in both SSc-ILD (61.4 ± 5.6% vs. 2.3 ± 0.6%, *p* = 0.002) and healthy controls (55.3 ± 2.7% vs. 1.16 ± 0.1%, *p* = 0.002). Raw data for panels **d**, **e** are shown in Supplemental Table S7. Statistical significance was determined initially with a Kruskal–Wallis across all subgroups followed by Mann–Whitney *U* tests for comparisons between groups if Kruskal–Wallis *p* < 0.05. *p < 0.05, **p < 0.01, ***p < 0.001, ****p < 0.0001

Marrack and colleagues previously showed that tBET was expressed in the CD21^lo^CD11c^+^ age-associated B cell populations [33], but tBET expression has not been evaluated in other CD21^lo/neg^ subsets. Flow cytometry was performed on a subset of 6 SSc-ILD and 6 healthy control samples to investigate the extent of tBET expression in the CD21^lo/neg^ population. tBET mean fluorescence intensity was markedly increased in CD21^lo/neg^ compared to CD21^+^ populations in both SSc-ILD (4110 ± 604 vs. 532 ± 90, *p* = 0.002) and healthy control (4019 ± 578 vs. 517 ± 27.3, *p* = 0.002), and most CD21^lo/neg^ B cells were tBET positive in both healthy control and SSc-ILD (Fig. 5b, e). In comparing the frequency of CD19^+^CD21^lo/neg−^CD11c^+^ in our CyTOF data to the frequency CD19^+^CD21^lo/neg^ tBET^+^ population identified by fluorescence cytometry, we found that these were the same for both healthy control (49.5 ± 3.8% vs. 55.3% ± 2.7%, *p* = 0.43) and SSc-ILD (64.07% ± 4.5% v 61.4% ± 5.6%, *p* = 0.70).

To determine if the decreased CD21 surface expression was a result of receptor localization or downregulation of protein expression, surface and internal expression of CD21 was also measured by flow cytometry. In both the healthy controls and SSc-ILD CD21^lo/neg^ subsets, no intracellular CD21 was detected, and the histogram matched that of the FMO contrast. In contrast, a small amount of intracellular CD21 staining was observed on the CD21^+^ subset (Fig. 5c, d).

### CD21^lo/neg^ B cells are present in the lung parenchyma of a patient with SSc‑ILD

**Fig. 6:**
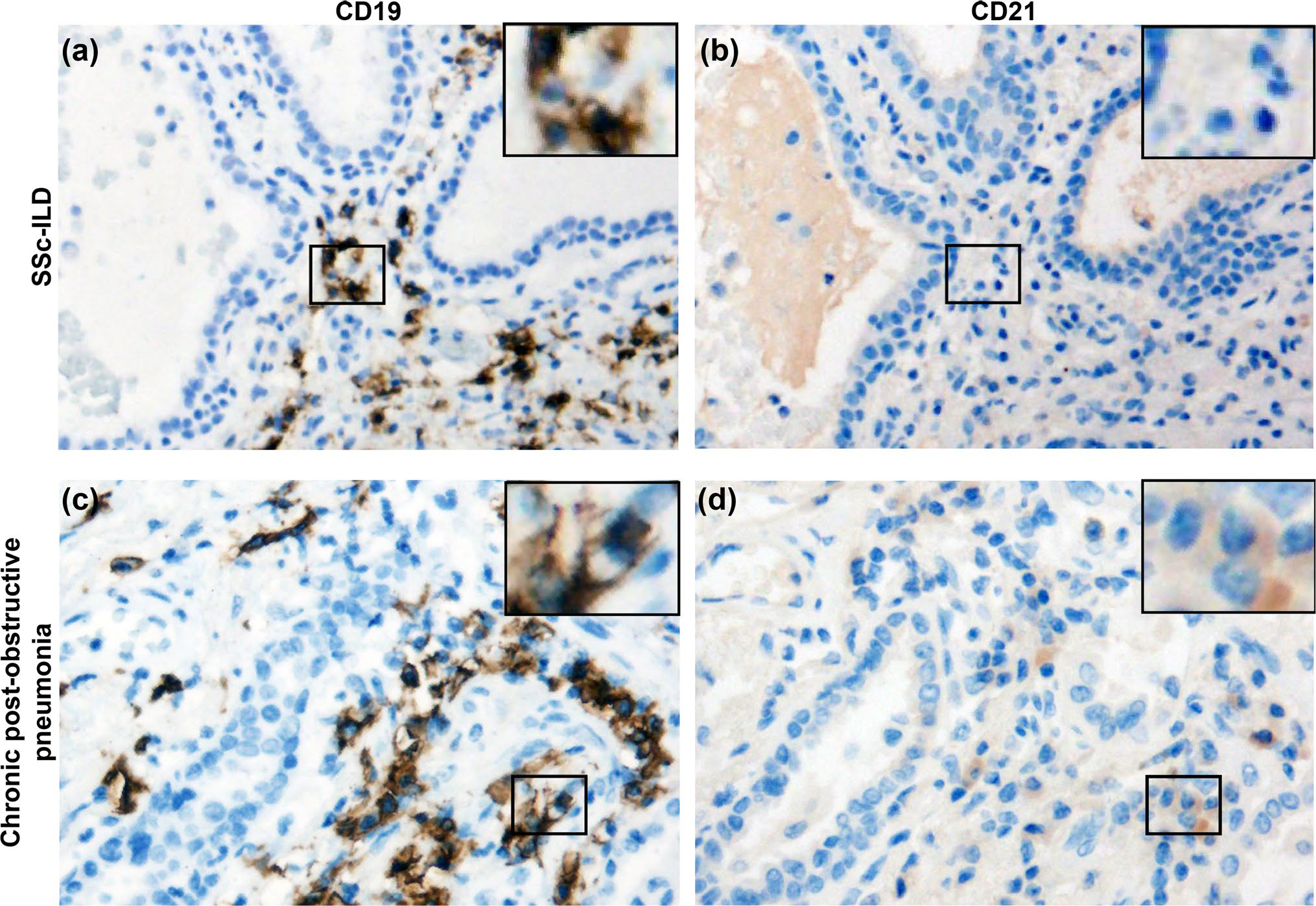
CD21^lo/neg^ B cells infiltrate the lung parenchyma in SSc-ILD but are rare in infectious pneumonia. Representative microphotographs are shown for an SSc-ILD lung explant stained for **a** CD19 and **b** CD21. Representative microphotographs for chronic post-obstructive pneumonia were also stained for **c** CD19 and **d** CD21 as an infection comparator. A positive control for panel B is shown in Figure S8

To evaluate the presence of CD21^lo/neg^ B cells in the lung parenchyma of SSc-ILD, IHC staining for CD19 and CD21 was performed in a SSc-ILD explant and a patient with chronic post-obstructive pneumonia (Fig. 6). The SSc-ILD patient had dense B cell infiltrate throughout all sections (Figs. 6a, S4), while the post-obstructive pneumonia parenchyma had only a single focus of B cell infiltrate (Fig. 6c). Within the lung parenchyma, most SSc-ILD B cells did not stain for CD21 (Fig. 6b), whereas most B cells in post-obstructive pneumonia were CD21^+^ (Fig. 6d). SSc-ILD germinal center B cell on the same slide stained for CD21 (Figure S5). These findings, although limited, suggest the abnormal circulating CD21^lo/neg^ population may localize to sites of pulmonary inflammation.

## Discussion

The primary results of these investigations can be summarized as follows: (1) CD21^lo/neg^ B cells are significantly increased in SSc-ILD but not SSc without ILD, (2) The CD21 ^lo/neg^ biaxial gate comprises several different cell subsets, all of which are increased in SSc-ILD, (3) The majority of CD21^lo/neg^ B cells are tBET positive, and (4) CD21^lo/neg^ B cells may infiltrate the lung parenchyma in SSc-ILD.

In addition to the novel findings we report, our results recapitulate previous B cell abnormalities observed in SSc. Similar to Sato [34], Forestier [35], and van der Kroef [36], we found that memory B cells were decreased. Like Dumoitier et al., we did not find a difference in total B or CD27^−^IgD^+^ naïve B cell frequency [37]. These findings give additional validity to our techniques and cohort.

The increased number of CD21^lo/neg^ B cells in SSc with ILD compared to those without was surprising. While the percent of CD21^lo/neg^ B cells was recently linked to SSc-PAH, this analysis did not evaluate the presence of ILD [38], which is a key cause of group III PAH [39, 40]. Similarly, Visentini and colleagues recently reported that CD21^lo/neg^ cells were associated digital ulcerations in SSc, but ILD was not evaluated in this cohort [41]. The overall importance of CD21^lo/neg^ B cells in autoreactivity has become increasingly clear in recent years [25]. The advent of checkpoint inhibitors for cancer immunotherapy facilitates studying the pathophysiological consequences of breaching immune tolerance in humans. In melanoma patients treated with combination anti-CTLA4 and anti-PD1 checkpoint blockade, an increase in CD21^lo/neg^ B cells could be detected after the first cycle of immunotherapy and increases in CD21^lo/neg^ B cells both preceded and correlated with frequency and severity of immune-related adverse events. These CD21^lo/neg^ B cells were also found to be more activated and have greater clonality than CD21^+^ B cells [42]. Other mechanistic studies have indicated that these CD21^lo/neg^ B cells might be potent activators of CD4^+^ T cells further driving tissue injury. In breast cancer, incubation of CD27^+^CD21^lo/med^ B cells with CD4 ^+^ T cells promoted Th1 responses and suppressed *T*_reg_ responses. CD27 + CD21^lo/med^ B cells were enriched in resected tumors and were thought to be critical mediators of anti-tumor immunity [43]. In mice, another population of CD21^lo/neg^ B cells has been implicated in the expansion of Th17 cells [44]. Although speculative, our findings support the notion that expansion of the CD21^lo/neg^ B cell compartment in SSc-ILD could drive local CD4^+^ lung inflammation. This B-T cell interplay might explain how rituximab has efficacy in SSc-ILD [23], which has previously been thought to be a T cell mediated process. Namely, elimination of B cells could decrease inflammatory CD4^+^ polarization, allow reconstitution of *T*_reg_ populations, and halt tissue inflammation and damage.

A second point of contention is whether the CD21^lo/neg^ phenotype arises from downregulation of CD21 protein or receptor internalization as ligand binding of CD21 leads to internalization of the receptor via its cytoplasmic tail [45] and greatly enhances the antigen presenting capabilities of B cells even in the absence of BCR stimulus [46]. Our flow cytometry results demonstrate that the CD21^lo/neg^ B cells in SSc-ILD do not have internalized CD21, which matches prior microarray analysis of CD21^lo/neg^ B cells in both rheumatoid arthritis and combined variable immunodeficiency [25, 42]. Additionally, histology visualizes proteins in the cell cytoplasm as well as surface, and no CD21 was detected in SSc-ILD lung tissue sections.

One major challenge in the treatment of interstitial lung disease is the accurate identification of CTD-ILD. Patients with CTD-ILD derive clear benefit from immunosuppressive treatments [47–49], while patients with idiopathic pulmonary fibrosis (IPF) are harmed by immunosuppression [50]. While protein biomarkers such as Krebs von den Lungen 6 and surfactant protein D are unable to distinguish IPF from CTD-ILD, cellular biomarkers of immune activation may more accurately predict CTD-ILD. Investigations of CD21^lo/neg^ frequency in other CTDs, IPF, and other chronic fibrosing lung diseases are warranted to investigate the potential of CD21^lo/neg^ B cells as specific biomarkers of SSc or SSc-spectrum-ILD.

We have demonstrated that patients with SSc-ILD have an increased frequency of circulating CD19^+^CD21^lo/neg^ cells and that CD19^+^CD21^lo/neg^ cells can be identified in the lung parenchyma. This study has several limitations, namely the small sample size that precludes multivariate analysis, the absence of longitudinal data, and the imperfect age matching between healthy controls and SSc patients. Additionally, the lack of disease activity and modified Rodnan’s skin scores prevented us from assessing of CD21^lo/neg^ frequency was associated with disease severity or extent of cutaneous involvement. Nonetheless, the recognition of a cellular subset that separates those SSc patients with and without ILD is potentially important. Additional work is needed to understand the mechanistic role of CD21^lo/neg^ B cells in SSc-ILD, their effectiveness as a potential biomarker for identifying SSc-ILD or other CTD-ILD, and the predictive value of CD21^lo/neg^ frequency at presentation on the future development of SSc-ILD.

## Supplementary Material

1749450_supmaterial

## Figures and Tables

**Table 1:** Patient demographics

	SSc patients (*n* = 48)	Healthy controls (*n* = 25)
Average age	59.4 ± 15.4	49.4 ± 11.9
Female gender	36 (75.0%)	15 (71.4%)
Race		
Caucasian	38 (79.2%)	23 (68.0%)
African American	6 (12.5%)	1 (4.0%)
Other	4 (8.3%)	1 (4.0%)
Average disease duration	9.2(4.0,14.6)	
Cutaneous involvement		
Limited	28 (61.4%)	
Diffuse	19 (39.6%)	
Interstitial lung disease	34 (70.8%)	
Average %FVC (*n* = 33)	71.7% ± 23.7%	
Average %DLCO (*n* = 30)	46.1% ± 22.7%	
Pulmonary hypertension	17 (35.4%)	
Serologic status		
+ ANA (*n* = 38)	36/38 (94.7%)	
Anti-centromere (*n* = 38)	11/38 (28.9%)	
Anti-Scl70 (*n* = 32)	12/32 (37.5%)	
Anti-RNA Pol III (*n* = 18)	4/18 (22.2%)	
Therapy at enrollment		
DMARD	12 (30.0%)	
No therapy	18 (37.5%)	
Meets 2013 classification criteria	48 (100%)	

Data reported as mean ± standard deviation except for disease duration, which is reported as the median with interquartile range

*FVC* forced vital capacity, *DLCO* diffusing capacity of the lung for carbon monoxide, *ANA* anti-nuclear antibodies, *DMARD* disease modifying anti-rheumatic drug and includes azathioprine, mycophenolate mofetil, leflunomide, methotrexate, tofacitinib, and tocilizumab

**Table 2:** Direct comparison of B cell frequencies in healthy controls and SSc with and without ILD

Population	Healthy control (*n* = 25)	SSc without ILD (*n* = 14)	SSc with ILD (*n* = 34)	Kruskal–Wallis *p* value	Post hoc Mann–Whitney *p* value		
					Healthy vs. SSc without ILD	Healthy vs. SSc with ILD	SSc without ILD vs. SSc with ILD
Total CD 19^+^	9.6 ± 0.9%	10.9 ± 1.3%	7.9 ± 1.2%	0.049	0.46	0.07	0.03
Naive^a^	65.1 ± 3.0%	75.7 ± 3.0%	65.9 ± 3.1%	0.06	–	–	–
Class-switched memory^b^	8.2 ± 0.9%	5.5 ± 1.2%	7.9 ± 1.1%	0.12	–	–	–
Non-class-switched memory^c^	13.1 ± 1.9%	3.7 ± 1.0%	3.4 ± 0.6%	< 0.0001	0.004	< 0.0001	0.36
CD21^lo/neg^	5.0 ± 0.5%	5.8 ± 0.9%	15.4 ± 13.3%	< 0.0001	0.55	< 0.0001	0.002
CD24^hi^CD38^hi^	5.6 ± 2.8%	9.3 ± 1.7%	4.6 ± 0.8%	0.003	0.03	0.048	0.002
CD27^−^IgD^−^ (DN B)	8.8 ± 1.1%	7.4 ± 0.9%	15.5 ± 1.9%	0.0009	0.39	0.003	0.001
CD21^+^ DN B	6.2 ± 0.9%	6.1 ± 0.6%	4.6 ± 0.5%	0.44	–	–	–
CD21^lo/neg^ DN B	2.4 ± 0.3%	2.7 ± 0.5%	9.2 ± 1.6%	< 0.0001	0.92	< 0.0001	0.0004
CD27^−^IgD^+^IgM^−^ (B_ND_)	3.1 ± 0.3%	4.1 ± 0.7%	4.2 ± 0.3%	0.15	–	–	–

All values expressed as cells/mL and reported as mean ± SEM

a CD27^−^/IgD^+^

b CD27^+^/IgM^−^/IgD^−^

c CD27^+^/IgM^+^

## Abbreviations

ACR: American College of Rheumatology
ANA: Anti-nuclear antibody
AZA: Azathioprine
BSA: Bovine serum albumin
CT: Computerized tomography
CTD: Connective tissue disease
CyTOF: Mass cytometry by time of flight
DLCO: Diffusion capacity of the lungs for carbon monoxide
DMARD: Disease modifying anti-rheumatic drug
EULAR: European league against rheumatism
FACS: Fluorescence-activated cell sorting
FMO: Fluorescence minus one
FVC: Forced vital capacity
IHC: Immunohistochemical
IL-6: Interleukin 6
ILD: Interstitial lung disease
MFI: Mean fluorescence intensity
MMF: Mycophenolate mofetil
PAH: Pulmonary arterial hypertension
PBS: Phosphate buffered saline
PBMC: Peripheral blood mononuclear cells
RF: Rheumatoid factor
RHC: Right heart catheterization
ROC: Reporter operator curve
RT: Room temperature
SSc: Systemic sclerosis
tSNE: T-distributed stochastic neighbor embedding
