# Supplementary material for: CD19 + CD21lo/neg cells are increased in systemic sclerosis-associated interstitial lung disease
Source: Clin Exp Med. Author manuscript; Available in PMC 2022 May 19. (PMC8828801; doi:10.1007/s10238-021-00745-5)
Supplement: Supplementary file 1 [file NIHMS1749450-supplement-1749450_supmaterial.docx]

**Supplemental Materials**

Supplemental Figures:	2

**Figure S1** Heatmap expression data for tSNE maps	2

**Figure S2** Comparison of healthy controls (n=25) and all SSc patients (n=48) for autoimmune prone populations identified in Figure 2.	3

**Figure S3** Additional CD21lo/neg subset analysis.	4

**Figure S4** CD21lo B cells infiltrate the lung parenchyma in SSc-ILD.	5

**Figure S5** CD21 positive control in SSc-ILD lung explant.	6

Supplemental Tables	7

**Table S1** Mass cytometry antibody clones	7

**Table S2** Fluorescence cytometry antibody clones	7

**Table S3** Markers utilized for B cell viSNE analysis	7

**Table S4** Detailed Clinical Phenotyping	8

**Table S5** Detailed Pulmonary Phenotyping	10

**Table S5** Raw data for Figure 4d-e.	12

# Supplemental Figures:

**Figure S1** Heatmap expression data for tSNE maps.  tSNE plots were generated from equal number of B cells concatenated from all patients and controls combined.  Heatmap expression data is shown for all markers used to generate tSNE maps with the exception of TIGIT and PD-1 that were not expressed on CD19^+^ cells.  Heatmap scale is generated for each channel across all files.

**Figure S2** Comparison of healthy controls (n=25) and all SSc patients (n=48) for autoimmune prone populations identified in Figure 2.  (a) There is no difference in the frequency of DN B (CD19+CD27-IgD-) cells in healthy controls or SSc patients (8.8 ± 1.1% v. 13.2 ± 1.5%, p=0.07).  (b) There is no difference in the frequency of CD24hiCD38hi transitional B cells in healthy controls or SSc patients (5.6 ± 0.6% v. 6.0 ± 0.8%, p=0.54). (c) There is no difference in the frequency of B_ND_ (CD19+CD27-IgD+IgM-) cells in healthy controls or SSc patients (3.2 ± 0.3% v. 4.2 ± 0.3%, p=0.06). (d) CD19+CD21^lo/neg^ cells are increased in SSc patients compared to healthy controls (12.6 ± 1.8% v. 5.0 ± 0.5%, p=0.0005).  Data reported as mean ± standard error of the mean.  Statistical significance was determined initially with a Kruskal-Wallis across all subgroups followed by Mann-Whitney U tests for comparisons between groups if Kruskal-Wallis p<0.05.  * p<0.05, ** p<0.01, ***p< 0.001, ****p<0.0001.

**Figure S3** Additional CD21lo/neg subset analysis. (a) Compared to healthy controls (n=25, 5.0 ± 0.5%), CD21^lo/neg^ frequency is increased in both limited cutaneous systemic sclerosis (lcSSc, n=29, 11.2 ±1.6%, p=0.002) and diffuse cutaneous systemic sclerosis (dcSSc, n=19, 14.7± 3.7%, p=0.005), but there is no difference between lcSSc and dcSSc (p=0.80).  (b) Compared to healthy controls (n=25), CD21^lo/neg^ frequency is increased in SSc patients with pulmonary hypertension (PH, n=17, 18.4 ± 3.6%, p<0.0001) and without PH (n=30, 9.5 ±1.7%, p=0.02), and patients with pulmonary hypertension had an increase in CD21^lo/neg^ frequency compared to patients without PH (p=0.02).  Pulmonary hypertension status was unknown for one patients.  (c) There was no difference in the frequency of CD21^lo/neg^ cells in PH patients or interstitial lung disease (ILD) patients without PH (12.5 ± 2.6%, p=0.23).  Data reported as mean ± standard error of the mean.  Statistical significance was determined initially with a Kruskal-Wallis across all subgroups followed by Mann-Whitney U tests for comparisons between groups if Kruskal-Wallis p<0.05.  * p<0.05, ** p<0.01, ***p< 0.001, ****p<0.0001.

**Figure S4** CD21lo B cells infiltrate the lung parenchyma in SSc-ILD.  Representative microphotographs from two different regions of a SSc-ILD lung explant stained for (A) CD19 and (B) CD21 at 10x power and (C) CD19 and (D) CD21 at 20x power.  

**Figure S5** CD21 positive control in SSc-ILD lung explant.  Detection of CD21 by IHC from the same section and slide as shown in Figure 5B demonstrating CD21 staining in a germinal center.  Dense CD21 staining is seen on the interior follicular dendritic cells with patchy CD21 staining visible in the surrounding B cells.

# Supplemental Tables

| **Table S1** Mass cytometry antibody clones |  |  |  |  |
| --- | --- | --- | --- | --- |
|  |  | **Metal** | **Target** | **Clone and Source** |
|  | Surface staining primary and direct antibodies |  |  |  |
|  |  | 89Y | CD45 | HI30 (Fluidigm) |
|  |  | 141Pr | CCR6 | G034E3 (Fluidigm |
|  |  | 142Nd | CD19 | HIB19 (Fluidigm) |
|  |  | 144Nd | CD11b | ICRF44 (Fluidigm) |
|  |  | 145Nd | CD4 | RPA-T4 (Fluidigm) |
|  |  | 146Nd | IgD | IA6-2 (Fluidigm) |
|  |  | 147Sm | CD11c | Bu15 (Fluidigm) |
|  |  | 148Nd | CD16 | 3G8 (Fluidigm) |
|  |  | 149Sm | CD127 | A019D5 (Fluidigm) |
|  |  | 150Nd | CD86 | IT2.2 (Fluidigm) |
|  |  | 151Eu | HLA-DR | G46-6 (Fluidigm) |
|  |  | 152Sm | CD21 | BL13 (Fluidigm) |
|  |  | 153Eu | CXCR5 | RF8B2 (Fluidigm) |
|  |  | 154Sm | TIGIT | MBSA43 (Fluidigm) |
|  |  | 155Gd | PD-1 | EH12.2H7 (Fluidigm) |
|  |  | 156Gd | CXCR3 | G025H7 (Fluidigm) |
|  |  | 158Gd | CCR4 | L291H4 (Fluidigm) |
|  |  | 159Tb | CCR7 | G043H7 (Fluidigm) |
|  |  | 160Gd | CD14 | M5E2 (Fluidigm) |
|  |  | 162Dy | CD27 | L128 (Fluidigm) |
|  |  | 163Dy | CD34 | 581 (Fluidigm) |
|  |  | 164Dy | CD45RO | UCHL1 (Fluidigm) |
|  |  | 166Er | CD24 | ML5 (Fluidigm) |
|  |  | 167Er | CD38 | HIT2 (Fluidigm) |
|  |  | 168Er | CD8 | SKI1 (Fluidigm) |
|  |  | 169Tm | CD25 | 2A3 (Fluidigm) |
|  |  | 170Er | CD3 | UCHT1 (Fluidigm) |
|  |  | 172Yb | IgM | MHM-88 (Fluidigm) |
|  |  | 173Yb | CXCR4 | 12G5 (Fluidigm) |
|  |  | --- | CX3CR1-biotin | 2A91 (Biolegend) |
|  |  | --- | CD28- APC | CD28.2 (Biolgend) |
|  | Surface staining secondary antibodies |  |  |  |
|  |  | 143Nd | Anti-biotin | ID4-C5 (Fluidigm) |
|  |  | 176Yb | Anti-APC | APC003 (Fluidigm) |
|  | Intracellular staining primary and direct antibodies |  |  |  |
|  |  | 171Yb | CD68 | Y1/82A (Fluidigm) |

| **Table S2** Fluorescence cytometry antibody clones |  |  |  |  |
| --- | --- | --- | --- | --- |
|  |  | **Fluorochrome** | **Target** | **Clone and Source** |
|  | Surface staining antibodies |  |  |  |
|  |  | AF700 | Live/dead |  |
|  |  | PE | CD21 (extracellular) | Bu32 (Biolegend) |
|  |  | BV510 | CD3 | OKT3 (Biolegend) |
|  |  | BV510 | CD14 | M5E2 (Biolegend) |
|  |  | BV510 | CD16 | 3G8 (Biolegend) |
|  |  | BUV395 | CD19 | SJ25C1 (BD) |
|  | Intracellular staining antibodies |  |  |  |
|  |  | PE-Dazzle | tBET | 4B10 (Biolegend) |
|  |  | FITC | CD21 (intracellular) | Bu32 (Biolegend) |

| **Table S3** Markers utilized for B cell viSNE analysis |  |  |  |  |  |  |  |
| --- | --- | --- | --- | --- | --- | --- | --- |
| CD45 | CCR6 | CD19 | CX3CR1 | CD11b | IgD | CD11c | CD16 |
| CD127 | CD86 | HLA-DR | CD21 | CXCR5 | TIGIT | PD-1 | CXCR3 |
| CCR4 | CCR7 | CD14 | CD27 | CD34 | CD45-RO | CD28 | CD24 |
| CD38 | CD25 | CD68 | IgM | CXCR4 |  |  |  |

|  | **Table S4.**  Patient Demographics for SSc Patients with and without ILD |  |  |  |
| --- | --- | --- | --- | --- |
|  |  | SSc-ILD (n=34) | SSc without ILD (n=14) | P value |
| Average Age |  | 61.1 ± 14.7 | 55.3 ± 15.7 | 0.37 |
| Female Gender |  | 25 (73.4%) | 11 (78.6%) | 1.00 |
| Race |  |  |  |  |
| Caucasian |  | 25 (73.5%) | 13 (92.9%) | 0.24 |
| African American |  | 6 (17.6%) | 0 (0%) | 0.16 |
| Other |  | 3 (8.8%) | 1 (7.1%) | 1.00 |
| Average Disease Duration |  | 11.6 ± 11.1 | 8.9 ± 5.5 | 0.75 |
| Cutaneous Involvement |  |  |  |  |
| Limited |  | 18 (52.9%) | 11 (78.6%) | 0.12 |
| Diffuse |  | 16 (47.1%) | 3 (21.4%) | 0.12 |
| Pulmonary Hypertension |  | 15 (44.1%) | 2 (14.3%) |  |
| SSc specific antibody |  |  |  |  |
| Anti-centromere |  | 4/27 (14.8%) | 7/11 (63.6%) | 0.005 |
| Anti-Scl70 |  | 9/22 (40.9%) | 3/10 (30.0%) | 0.70 |
| Anti-RNA Pol III |  | 3/14 (21.4%) | 1/4 (25.0%) | 1.00 |
| Therapy at enrollment |  |  |  |  |
| DMARD |  | 18 (52.9%) | 1 (7.1%) | 0.004 |
| No therapy |  | 11 (32.3%) | 7 (50%) | 0.33 |
| Meets 2013 Classification Criteria |  | 34 (100%) | 14 (100%) | 1.0 |
| Data reported as mean ± standard deviation except for disease duration, which is reported as the median with interquartile range.  Statistical significance determined by Mann-Whitney U tests or Fischer’s exact tests, as appropriate. Abbreviations: FVC = forced vital capacity, DLCO = diffusing capacity of the lung for carbon monoxide, ANA = anti-nuclear antibodies, DMARD = disease modifying anti-rheumatic drug and includes azathioprine, mycophenolate mofetil, leflunomide, methotrexate, tofacitinib, and tocilizumab. |  |  |  |  |

| **Table S5** Detailed Clinical Phenotyping |  |  |  |  |  |  |  |  |  |
| --- | --- | --- | --- | --- | --- | --- | --- | --- | --- |
| Subject ID | Gender | Age (yrs) | Disease Duration (yrs) | Immunomodulatory agents within 6 mos of enrollment | Prior Immunosuppressive Medications | Diffuse Cutaneous SSc | Pulmonary hypertension | Interstitial Lung Disease | SSc related Autoantibody |
| 1 | F | 62.8 | 8.8 | None | None | No | Yes | Yes | None |
| 2 | F | 67.0 | 16.8 | None | MMF | No | No | No | Centromere |
| 3 | F | 60.3 | 11.8 | HCQ | None | No | Yes | Yes | None |
| 4 | F | 61.6 | 10.8 | AZA | None | Yes | Yes | Yes | unknown |
| 5 | F | 48.5 | 5.6 | MMF | P < 10mg, CYC, MTX | Yes | No | Yes | Scl70 |
| 6 | M | 66.0 | 7.8 | None | None | No | No | Yes | Scl70 |
| 7 | F | 57.2 | 6.8 | None | P<10, MMF, | No | Yes | No | Scl70 |
| 8 | F | 81.7 | 52.8 | None | None | No | Yes | Yes | None |
| 9 | F | 66.0 | 4.8 | P5, leflunamide | ND | Yes | Yes | Yes | None |
| 10 | M | 64.4 | 9.8 | HCQ | P<10, AZA | No | No | No | Scl70 |
| 11 | F | 40.9 | 5.9 | None | None | No | No | Yes | Scl70 |
| 12 | F | 53.4 | 9.9 | None | None | No | No | No | Centromere |
| 13 | F | 43.9 | 12.0 | P5 | MTX | No | No | No | unknown |
| 14 | M | 62.3 | 14.0 | MTX | None | Yes | No | No | unknown |
| 15 | F | 70.9 | 33.0 | MMF | P10-30, CYC, AZA, CYA, MTX, | Yes | Yes | Yes | unknown |
| 16 | F | 76.7 | 4.0 | MTX/HCQ | P <10 | No | Yes | Yes | Centromere |
| 17 | F | 76.9 | 21.1 | P5 | ND | No | Yes | Yes | unknown |
| 18 | F | 68.2 | 18.1 | HCQ | MMF | No | No | No | Centromere |
| 19 | M | 72.8 | 15.1 | MMF/P5 | AZA | No | Yes | Yes | None |
| 20 | F | 19.8 | 1.4 | HCQ | None | Yes | No | No | Scl70 |
| 21 | F | 66.7 | 9.1 | P2 | None | No | Yes | No | unknown |
| 22 | F | 60.0 | 1.1 | MMF | None | No | Yes | Yes | unknown |
| 23 | F | 62.3 | 14.6 | HCQ | AZA | No | Yes | Yes | unknown |
| 24 | F | 73.6 | 4.1 | None | MTX | Yes | No | Yes | RNApol3 |
| 25 | F | 21.1 | 5.1 | None | None | No | No | No | Centromere |
| 26 | M | 80.2 | 0.8 | None | None | Yes | Yes | Yes | Scl70 |
| 27 | F | 52.3 | 2.2 | None | None | No | No | Yes | Scl70 |
| 28 | M | 66.6 | 11.2 | P 5 | D-penicillamine | Yes | Yes | Yes | Scl70 |
| 29 | F | 59.9 | 17.2 | None | None | No | No | Yes | None |
| 30 | M | 55.8 | 4.4 | AZA/P5 | CYC | No | No | Yes | Pm/Scl |
| 31 | F | 58.5 | 34.2 | None | D-penicillamine | No | No | Yes | None |
| 32 | F | 71.4 | 9.2 | Leflunamide/Tofacitinb | P<10, MTX, ADA, CER | No | No | Yes | Centromere |
| 33 | F | 59.5 | 9.5 | D-penicillamine | None | No | No | No | Centromere |
| 34 | M | 54.2 | 13.7 | MMF | None | No | No | Yes | None |
| 35 | M | 60.6 | 17.2 | None | CYC, MTX | Yes | Yes | Yes | Unknown |
| 36 | F | 62.9 | 1.7 | None | None | No | No | No | Centromere |
| 37 | M | 56.0 | 2.0 | None | None | Yes | No | No | RNApol3 |
| 38 | F | 18.7 | 2.3 | MMF, HCQ | None | Yes | No | Yes | None |
| 39 | F | 67.6 | 1.9 | MMF/HCQ | P<10, MTX | No | No | Yes | None |
| 40 | F | 71.4 | unknown | None | None | No | No | No | Centromere |
| 41 | F | 86.0 | 23.6 | HCQ | P<10 | Yes | Yes | Yes | Centromere |
| 32 | F | 47.8 | 14.8 | TOZ, P6 | P10-30, MMF | Yes | No | Yes | Scl70 |
| 43 | F | 37.2 | 6.8 | MMF | None | Yes | No | Yes | RNApol3 |
| 44 | F | 71.3 | 13.8 | None | None | No | No | Yes | Centromere |
| 45 | M | 78.9 | 2.0 | MTX | None | Yes | No | Yes | Scl70 |
| 46 | F | 39.5 | 11.1 | MMF | MTX, IVIG | Yes | Unknown | Yes | Pm/Scl |
| 47 | F | 51.0 | 2.6 | MMF | None | Yes | No | Yes | RNApol3 |
| 48 | M | 38.4 | 3.2 | MMF | MTX | Yes | No | Yes | Scl70 |

| **Table S6** Detailed Pulmonary Phenotyping |  |  |  |  |  |  |  |  |  |  |  |  |  |  |  |  |  |  |  |
| --- | --- | --- | --- | --- | --- | --- | --- | --- | --- | --- | --- | --- | --- | --- | --- | --- | --- | --- | --- |
| Subject ID | Supplemental Oxygen at enrollment | Antifibrotic therapy ever | PH evaluation |  |  |  |  |  |  | ILD evaluation |  |  |  |  |  |  |  |  |  |
|  |  |  | PH y/n | Time from enroll^†^ (months) | Echo RVSP (mmHg) | Time of RHC from enroll^† ^(months) | mPAP | PCWP | PVR | SSc-ILD y/n | CT findings based on radiology report |  |  |  |  |  | PFTs |  |  |
|  |  |  |  |  |  |  |  |  |  |  | Time from enroll (months)^†^ | No ILD | GGOs | Reticulations | BC | HC | Time enroll (months)^†^ | Worst FVC | Worst DLCO |
| 1 | Yes | No | Yes | -3.1 | 85 | -2.2 | 46 | 5 | 9.9 | Yes | -2.0 |  |  | X | X | X | -1.8 | 66 | 13 |
| 2 | No | No | No | -45.2 | 30 | -69.7 | 21 | 11 | 2.2 | No | -5.8 | X |  |  |  |  | -2.3 | 100 | 58 |
| 3 | Yes | No | Yes | 0.0 | >100 | -1.9 | 47 | 10 | 8.2 | Yes | -13.1 |  | X | X | X | X | -1.9 | 70 | 21 |
| 4 | No | No | Yes | 0.0 | 24 | -66.7 | 25 | 11 | 4.2 | Yes | -68.3 |  | X | X | X |  | 0.0 | 63 | 61 |
| 5 | No | No | No | 1.8 | “normal” | --- | --- | --- | --- | Yes | -55.6 |  | X | X |  |  | 1.8 | 58 | 53 |
| 6 | No | No | No | -31.1 | 38 | -30.1 | 19 | 8 | 1.5 | Yes | -4.4 |  | X |  |  |  | 0.0 | 70 | 61 |
| 7 | No | No | Yes | -5.3 | “normal” | -76.2 | 32 | 10 | NR | No | -3.5 | X |  |  |  |  | 0.0 | 31 | 8 |
| 8 | Yes | No | Yes | -0.9 | 75 | -0.7 | 42 | 2 | 15.8 | Yes | --- | --- | --- | --- | --- | --- | -0.8 | 37 | 23 |
| 9 | Yes | No | Yes | 0.0 | 63 | -19.5 | 44 | 10 | 14.1 | Yes | -9.6 |  | X | X | X | X | 0.0 | 22 | NR |
| 10 | No | No | No | -43.6 | 46 | -42.2 | 9 | 3 | NR | No | --- | --- | --- | --- | --- | --- | -4.2 | 89 | 84 |
| 11 | Yes | No | No | -2.3 | 33 | -5.0 | 19 | 7 | 3.0 | Yes | -5.1 |  | X | X | X | X | -2.3 | 31 | NR |
| 12 | No | No | No | -11.3 | “normal” | --- | --- | --- | --- | No | --- | --- | --- | --- | --- | --- | -11.0 | 120 | 83 |
| 13 | No | No | No | -4.2 | “normal” | --- | --- | --- | --- | No | -117.5 | X |  |  |  |  | -13.3 | 103 | 60 |
| 14 | No | No | No | -44.8 | “normal” | --- | --- | --- | --- | Yes | 34.3 |  |  | X |  |  | -44.8 | 70 | 81 |
| 15 | No | No | Yes | -11.5 | 51 | -10.8 | 28 | 13 | 3.9 | Yes | -11.2 |  |  | X | X | X | -11.2 | 60 | 41 |
| 16 | No | No | Yes | 0.0 | 57 | -6.8 | 36 | 3 | 7.3 | Yes | -31.6^‡^ |  |  |  |  |  | -32.0 | 119 | 20 |
| 17 | Yes | No | Yes | -6.2 | 75 | -16.8 | 39 | 14 | 6.6 | Yes | -108.0 |  | X | X |  | X | -18.4 | 80 | 29 |
| 18 | No | No | No | -37.1 | NR | --- | --- | --- | --- | No | -16.3 |  |  |  |  |  | -19.4 | 112 | NR |
| 19 | No | No | Yes | -12.4 | 40 | -30.9 | 26 | 8 | 3.62 | Yes | -50.2 |  | X | X | X |  | -6.3 | 61 | 26 |
| 20 | No | No | No | -0.7 | NR | --- | --- | --- | --- | No | -0.7 | X |  |  |  |  | -0.7 | 112 | 110 |
| 21 | No | No | Yes | -12.1 | 35 | -85.4 | 26 | 14 | NR | No | --- | --- | --- | --- | --- | --- | -92.0 | 87 | 72 |
| 22 | No | No | Yes | 6.0 | 65 | --- | --- | --- | --- | Yes | -13.9 |  |  | X | X |  | 0.0 | 56 | NR |
| 23 | Yes | No | Yes | 0.0 | 73 | 2.0 | 41 | 7 | 5.8 | Yes | -88.4^⁑^ |  |  |  |  |  | -88.9 | 72 | 33 |
| 24 | No | No | No | -0.9 | 20 | --- | --- | --- | --- | Yes | 1.0 |  | X | X |  |  | 2.5 | 90 | 25 |
| 25 | No | No | No | -6.7 | NR | --- | --- | --- | --- | No | -55.6 | X |  |  |  |  | -4.5 | 92 | 52 |
| 26 | No | No | Yes | -0.1 | 25 | -0.1 | 35 | 16 | 4.9 | Yes | 0.0 |  | X | X | X |  | 0.0 | 82 | 36 |
| 27 | No | No | No | 9.0 | NR | --- | --- | --- | --- | Yes | -1.1 |  | X | X |  |  | 0.0 | 111 | 107 |
| 28 | Yes | No | Yes | -72.2 | NR | -72.2 | 33 | 16 | 2.9 | Yes | -72.3 |  | X |  | X | X | -72.3 | 40 | 40 |
| 29 | No | No | No | 1.0 | 27 | --- | --- | --- | --- | Yes | 1.0 |  |  | X |  |  | 4.5 | 73 | 61 |
| 30 | Yes | No | No | 3.0 | NR | --- | --- | --- | --- | Yes | -54.1 |  |  | X | X |  | -49.4 | 72 | 42 |
| 31 | No | No | No | -94.6 | 25 | --- | --- | --- | --- | Yes^⁂^ | --- | --- | --- | --- | --- | --- | -171.3 | 77 | 78 |
| 32 | No | No | No | -15.1 | 30-40 | -23.4 | 15 | 4 | 2.4 | Yes | -11.2 |  | X |  | X |  | -24.0 | 76 | 68 |
| 33 | No | No | No | -97.0 | 24 | --- | --- | --- | --- | No | --- | --- | --- | --- | --- | --- | -106.5 | 99 | 67 |
| 34 | No | No | No | 0.2 | NR | --- | --- | --- | --- | Yes | 129.6 |  | X | X | X |  | -11.1 | 87 | 51 |
| 35 | No | No | Yes | 9.1 | NR | 9.6 | 24 | 3 | 4.6 | No | --- | --- | --- | --- | --- | --- | 9.1 | 103 | 43 |
| 36 | No | No | No | 9.0 | 15 | --- | --- | --- | --- | No | --- | --- | ---- | --- | --- | --- | -4.0 | 115 | 88 |
| 37 | No | No | No | -0.5 | 30 | --- | --- | --- | --- | No | -0.5 | X |  |  |  |  | -0.5 | 89 | 84 |
| 38 | No | No | No | 0.2 | 14 | --- | --- | --- | --- | Yes | 2.0 |  | X |  |  |  | 3.6 | 89 | 75 |
| 39 | No | No | No | -9.8 | 24 | --- | --- | --- | --- | Yes | -9.8 |  |  | X |  |  | -2.1 | 86 | 93 |
| 40 | No | No | No | -27.3 | 16 | --- | --- | --- | --- | No | -100.0 | X |  |  |  |  | -27.3 | 95 | 61 |
| 41 | No | No | Yes | 4.0 | 62 | --- | --- | --- | --- | Yes^‡^ | -54.0 |  |  |  |  |  | -13.0 | 101 | 41 |
| 32 | Yes | No | No | -2.3 | 44 | -38.3 | 16 | 9 | 1.6 | Yes | -5.3 |  |  | X | X | X | -2.3 | 27 | 7 |
| 43 | No | No | No | -11.1 | 40 | --- | --- | --- | --- | Yes | -8.2 |  |  | X |  |  | -8.2 | 73 | 65 |
| 44 | No | No | No | -17.1 | 20 | --- | --- | --- | --- | Yes | 0.0 |  | X | X |  |  | 1.1 | 111 | 44 |
| 45 | Yes | No | No | 0.0 | 38 | --- | --- | --- | --- | Yes | 0.0 |  |  | X |  |  | ---§ | ---§ | ND |
| 46 | No | No | Unknown | --- | --- | --- | --- | --- | --- | Yes | -45.3 |  | X | X |  |  | -11.3 | 79 | 46 |
| 47 | No | No | No | -3.3 | 35 | -4.7 | 29 | 20 | 1.7 | Yes | -30.5 |  | X |  |  |  | -30.6 | 59 | 43 |
| 48 | No | No | No | -0.2 | NR | 0.0 | 24 | 15 | 1.6 | Yes | -3.8 |  | X | X | X |  | 0.0 | 60 | 38 |
| †Negative time periods indicate the test was performed before blood collection, positive values indicate the test was performed after blood collection ‡Per radiology report, “bibasilar scarring” ^⁂^Clinic notes state prior radiographic ILD ^⁑^numerous scattered micronodules with volume loss §Uninsured patient presenting to the ICU with SSc renal crisis who died and had never received PFTs --- indicates missing data Abbreviations: BC=bronchiectasis, DLCO=diffusion capacity of the lungs for carbon monoxide, FVC=forced vital capacity, GGOs=ground glass opacities, HC=honeycombing, ILD=interstitial lung disease, NR=not reported, mPAP=mean pulmonary artery pressure, PCWP=pulmonary capillary wedge pressure, PFTs=pulmonary function tests, PH=pulmonary hypertension, PVR=pulmonary vascular resistance, RHC=right heart cath, RVSP=right ventricular systolic function, |  |  |  |  |  |  |  |  |  |  |  |  |  |  |  |  |  |  |  |

## **Table S7** Raw data for Figure 5d-e.

| Intracellular CD21 MFI |  |  |  | %tBET positive |  |  |  |
| --- | --- | --- | --- | --- | --- | --- | --- |
| HC CD21^hi^ | SSc-ILD CD21^hi^ | HC CD21^lo^ | SSc-ILD CD21^lo^ | HC CD21^hi^ | SSc-ILD CD21^hi^ | HC CD21^lo^ | SSc-ILD CD21^lo^ |
| 340 | 245 | 13.1 | 13.1 | 0.867 | 1.3 | 51.7 | 54.5 |
| 304 | 266 | 31.2 | 7.61 | 1.12 | 5.22 | 51.3 | 78.4 |
| 240 | 245 | 14.2 | 18.3 | 1.02 | 1.14 | 50.9 | 55.6 |
| 384 | 340 | 9.79 | 19.4 | 1.35 | 1.85 | 68.6 | 78.2 |
| 334 | 194 | 10.9 | 31 | 1.64 | 3.03 | 55.2 | 56.3 |
| 234 | 311 | 5.66 | 42.8 | 0.962 | 1.28 | 54.3 | 45.4 |
| Abbreviations: HC=healthy control, MFI=mean fluorescent intensity, SSc-ILD=systemic sclerosis associated interstitial lung disease |  |  |  |  |  |  |  |
